# Hydrogen sulfide ameliorates chronic renal failure in rats by inhibiting apoptosis and inflammation through ROS/MAPK and NF-*κ*B signaling pathways

**DOI:** 10.1038/s41598-017-00557-2

**Published:** 2017-03-28

**Authors:** Dongdong Wu, Ning Luo, Lianqu Wang, Zhijun Zhao, Hongmin Bu, Guoliang Xu, Yongjun Yan, Xinping Che, Zhiling Jiao, Tengfu Zhao, Jingtao Chen, Ailing Ji, Yanzhang Li, Garrick D. Lee

**Affiliations:** 1 Henan University School of Medicine, Kaifeng, 475004 Henan China; 2 The First Affiliated Hospital of Henan University, Kaifeng, 475001 Henan China; 3 Luohe Medical College, Luohe, 462002 Henan China

## Abstract

Chronic renal failure (CRF) is a major public health problem worldwide. Hydrogen sulfide (H_2_S) plays important roles in renal physiological and pathophysiological processes. However, whether H_2_S could protect against CRF in rats remains unclear. In this study, we found that H_2_S alleviated gentamicin-induced nephrotoxicity by reducing reactive oxygen species (ROS)-mediated apoptosis in normal rat kidney-52E cells. We demonstrated that H_2_S significantly improved the kidney structure and function of CRF rats. We found that H_2_S decreased the protein levels of Bax, Caspase-3, and Cleaved-caspase-3, but increased the expression of Bcl-2. Treatment with H_2_S reduced the levels of malondialdehyde and ROS and increased the activities of superoxide dismutase and glutathione peroxidase. H_2_S significantly abolished the phosphorylation of extracellular signal-regulated protein kinase 1/2, c-Jun N-terminal kinase, and p38 in the kidney of CRF rats. Furthermore, H_2_S decreased the expression levels of tumor necrosis factor-α, interleukin (IL)-6, IL-10, and monocyte chemoattractant protein-1, as well as the protein levels of p50, p65, and p-p65 in the kidney of CRF rats. In conclusion, H_2_S could ameliorate adenine-induced CRF in rats by inhibiting apoptosis and inflammation through ROS/mitogen-activated protein kinase and nuclear factor-kappa B signaling pathways.

## Introduction

As the incidence of chronic renal failure (CRF) increases at an alarming rate, CRF has been considered a major public health problem worldwide^1^. CRF is a syndrome characterized by progressive and irreversible deterioration of renal function^2^. In humans, CRF is mainly caused by hypertension, glomerulonephritis, and diabetes mellitus^3, 4^. This condition is made worse by a deterioration in nutrition level caused by accumulation of uremic toxins and reduced food intake, which could be attributed to many factors, such as gastrointestinal congestion, loss of appetite, and reduced glomerular filtration rate^4–6^. Compared to patients with other chronic diseases, patients with CRF tend to require longer and more frequent hospitalizations which are associated with higher morbidity and mortality^7^. Therefore, it is urgent to develop alternative medicines and novel therapies for the treatment of CRF.

Hydrogen sulfide (H_2_S) has recently been identified as the third endogenous gaseous transmitter that is enzymatically synthesized by cystathionine γ-lyase (CSE), cystathionine β-synthase (CBS), and 3-mercaptopyruvate sulfurtransferase (3-MST) in mammalian tissues^8, 9^. These enzymes have been identified in the kidney and are responsible for endogenous renal H_2_S production^10–12^. An increasing number of studies indicate that H_2_S plays an important role in renal physiology and pathology^11, 13, 14^. The physiological level of H_2_S leads to vasodilation and increases glomerular filtration rate and renal blood flow, which causes an indirect increase of the urinary excretion of K^+^ and Na^+^^13^. Under pathological conditions, H_2_S exerts the protective role in a number of experimental models of renal disease, including obstructive nephropathy^11^, renal ischemia-reperfusion injury^15^, and diabetic nephropathy^16^. Recent studies demonstrated that H_2_S is significantly lower in plasma and tissues of uremic patients and 5/6 nephrectomy rats^17–19^. Considering the beneficial effects of H_2_S in renal physiology and pathology, we hypothesize that the application of exogenous H_2_S may effectively protect against CRF.

Gentamicin (GEN), an aminoglycoside antibiotic, plays an important role in the treatment of a wide range of gram-negative bacterial infections. However, nephrotoxicity is considered as its major side effect, which seriously limits its clinical use^20, 21^. GEN has been widely used in *in vitro* models through inducing nephrotoxicity, such as normal rat kidney (NRK)-52E cells^20–22^. In this study, we investigated the effects of H_2_S on the nephrotoxicity induced by GEN in NRK-52E cells. The adenine-induced CRF model in rats is a standard method for inducing metabolic abnormalities closely resembling those observed in uremic patients^23, 24^. Therefore, a rat model of adenine-induced CRF was used in the present study. We also investigated the effects and mechanisms of H_2_S on adenine-induced CRF in rats.

## Materials and Methods

### Cell culture

NRK-52E cells were obtained from iCell Bioscience Inc. (Shanghai, China) and maintained in Dulbecco’s modified Eagle’s medium (DMEM)/F12 medium supplemented with 10% fetal calf serum, 100 µg/ml streptomycin, and 100 U/ml penicillin. Cells were cultured in a humidified incubator with 5% CO_2_ and 95% air at 37 °C. Confluent NRK-52E cells were transferred to serum-free DMEM/F12 medium for overnight starvation before each experiment. NRK-52E cells were incubated with 3 mM GEN for 24 h to induce nephrotoxicity^25^. The cells were divided into three groups: Control group, GEN group, and GEN+H_2_S group. The control and GEN groups were treated with phosphate-buffered saline (PBS) and the GEN+H_2_S group was treated with 100 µM NaHS (an H_2_S donor, dissolved in PBS). Treatments with PBS and NaHS were concomitant to GEN-induced nephrotoxicity for 24 h.

### Cell growth assay

For 5-ethynyl-2′-deoxyuridine (EdU) incorporation assay, the proliferating cells were examined using the Cell-Light EdU Apollo 567 *In Vitro* Imaging Kit (RiboBio, Guangzhou, Guangdong, China). Briefly, after incubation with 10 mM EdU for 2 h, NRK-52E cells were fixed with 4% paraformaldehyde, permeabilized with 0.3% Triton X-100 and stained with the fluorescent dyes. 4′,6-diamidino-2-phenylindole (DAPI) (5 mg/ml) was used to stain the cell nuclei for 10 min at room temperature. Then the cells were visualized under a fluorescent microscope (Eclipse Ti, Nikon, Melville, NY, USA) from five random fields. Cell proliferation rate = (EdU-positive cells)/(total number of cells) × 100%. Cell growth was also measured using the CellTiter 96 AQ_ueous_ One Solution Cell Proliferation Assay kit (MTS; Promega, Madison, WI, USA) according to the manufacturer’s protocols.

### Detection of intracellular reactive oxygen species (ROS)

Intracellular ROS generation was measured by using a 2′,7′-dichlorofluorescin diacetate (DCF-DA)-Cellular Reactive Oxygen Species Detection Assay Kit (Beyotime Institute of Biotechnology, Shanghai, China). Cells were incubated with 10 μM DCF-DA for 30 min at 37 °C and washed three times with PBS. The fluorescence was observed by a fluorescent microscope (Eclipse Ti, Nikon, Melville, NY, USA) from five random fields and measured by ImageJ software (National Institutes of Health, Bethesda, MD, USA).

### Cellular apoptosis analysis

Cellular apoptosis was analyzed by performing a terminal deoxynucleotidyl transferase-mediated dUTP nick end labeling (TUNEL) assay using an *in situ* cell death detection kit (Beyotime Institute of Biotechnology, Shanghai, China) following the manufacturer’s instructions. After 4% paraformaldehyde fixation and 0.1% Triton X-100 permeabilization, cells were incubated with 50 μl TUNEL reaction mixture for 60 min at 37 °C in the dark and then rinsed with PBS three times. Then after a 10 min DAPI (5 mg/ml) counterstain at room temperature, cells were photographed with a fluorescent microscope (Eclipse Ti, Nikon, Melville, NY, USA) from five random fields. The apoptotic index = (positively stained apoptotic cells)/(total number of cells) × 100%.

### Ethics statement

Animal experiments were approved by the Committee of Medical Ethics and Welfare for Experimental Animals of Henan University School of Medicine in compliance with the Experimental Animal Regulations formulated by the National Science and Technology Commission, China. Animal experiments were conducted in accordance with the committee’s approved guidelines.

### Animals

Twenty-four male Wistar rats (7–9 weeks old), initially weighing 180–220 g, were purchased from the Nanjing Biomedical Research Institute of Nanjing University (Jiangsu, China). Rats were housed in individual ventilated cages under standard temperature (22 ± 2 °C), humidity (50–60%), and light conditions (12-hour light/dark cycle) with food and water *ad libitum*. Rats were allowed to acclimatize to new surroundings for 1 week before the experiment began. CRF was induced with 0.2% adenine mixed with powdered food for 4 weeks. A normal renal function control group was also allocated. The rats from control and CRF groups received an intraperitoneal (i.p.) injection of saline and the rats from the CRF+H_2_S group received an i.p. injection of NaHS (100 μmol/kg/day, dissolved in saline)^26^. Treatments with saline and NaHS were concomitant to adenine-induced CRF for 4 weeks. During the treatment periods, the rats were weighed weekly and the food intake, water intake, and urine volume were measured in 24 h. At the end of experiments, the rats were killed and the plasma was collected. Tissues were rapidly removed, weighed and thoroughly washed with ice-cold saline. Then the tissues were frozen in liquid nitrogen or immersed in 4% neutral buffered formalin or embedded in FSC 22 frozen section compound (Leica, Buffalo Grove, IL, USA). Plasma samples and frozen tissues were stored at −80 °C.

### Histological analysis

The renal tissues were fixed in formalin, embedded in paraffin, and cut into 5-μm-thick sections which were then stained with hematoxylin and eosin (HE) and Masson’s trichrome (MT). The histopathological score was obtained based on the loss of brush border, grading of tubular necrosis, tubular dilatation, and cast formation in six randomly chosen, non-overlapping fields as follows: 0 (none), 1 (≤10%), 2 (11–25%), 3 (26–45%), 4 (46–75%), and 5 (≥76%)^27^. The extent of renal interstitial fibrosis (RIF) was scored from 0 to 3 as follows: 0 = absent, 1 = less than 25% of the area, 2 = 25–50% of the area, and 3 = more than 50% of the area. The RIF index was obtained by the following formula: RIF index = (0 × *n* 0 + 1 × *n* 1 + 2 × *n* 2 + 3 × *n* 3)/(*n* 0 + *n* 1 + *n* 2 + *n* 3) × 100%^28^. All specimens were anonymized and evaluated in a blinded manner. The sections were observed with an Olympus BX51 microscope (Olympus, Tokyo, Japan) and analyzed by ImageJ software (National Institutes of Health, Bethesda, MD, USA).

### Biochemical analysis

Blood urea nitrogen (BUN), creatinine (Cre), and urinary protein (UP) were measured using Beckman Coulter AU5800 (Beckman Coulter Inc., Brea, CA, USA). The levels of white blood cell (WBC), red blood cell (RBC), hemoglobin (HGB), and hematocrit (HCT) were determined by Mindray BC-6900 auto hematology analyzer (Mindray, Shenzhen, Guangdong, China). Monocyte chemoattractant protein (MCP)-1, tumor necrosis factor (TNF)-α, interleukin (IL)-6, and IL-10 in kidney tissues were determined using commercial ELISA kits (Elabscience, Wuhan, Hubei, China) according to the manufacturer’s protocols.

### Western blot analysis

Renal tissues were homogenized in RIPA lysis buffer (Sigma, St. Louis, MO, USA). Protein concentrations of the homogenates were measured by the BCA protein assay kit (Beyotime Institute of Biotechnology, Shanghai, China). The extracted proteins (50 μg) were separated on SDS-PAGE gel and transferred to a PVDF-nitrocellulose membrane. After blocking, the membranes were incubated with primary antibodies to detect the target proteins. Anti-extracellular signal-regulated protein kinase 1/2 (ERK1/2), anti-phospho (p)-ERK1/2 (Thr202/Tyr204), anti-c-Jun N-terminal kinase (JNK), anti-p-JNK (Thr183/Tyr185), anti-p38, anti-p-p38 (Thr180/Tyr182), anti-p50, anti-p65, and anti-p-p65 (Ser536) antibodies were purchased from Cell Signaling Technology (Danvers, MA, USA). Anti-Bax, anti-Bcl-2, anti-Caspase-3, anti-Cleaved Caspase-3, and anti-β-actin antibodies were purchased from ProteinTech (Chicago, IL, USA). The horseradish peroxidase-conjugated secondary antibody was purchased from Cell Signaling Technology. The reaction was visualized using an enhanced chemiluminescence system (Thermo Fisher Scientific, Rockford, IL, USA). The bands were quantified by densitometry using ImageJ software.

### Measurement of oxidative stress products

The kidney tissues were placed in cold physiological saline, homogenized with a homogenizer machine (Scientz Biotechnology Co., Ltd., Ningbo, Zhejiang, China), and then centrifuged at 1000 g for 10 min to produce the supernatant fluid. The levels of malondialdehvde (MDA) and ROS, as well as the activities of glutathione peroxidase (GSH-Px) and superoxide dismutase (SOD) were measured using commercial kits (Nanjing Jiancheng Bioengineering Institute, Nanjing, Jiangsu, China) according to the manufacturer’s instructions.

### Statistical analysis

All results were presented as the mean ± standard error of the mean (SEM). Statistical differences were analyzed by one-way analysis of variance (ANOVA) using SPSS 17.0 software, followed by LSD post hoc test. A *P* value of less than 0.05 was considered to be statistically significant.

## Results

### H_2_S relieves GEN-induced cytotoxicity in NRK-52E cells

**Figure 1:**
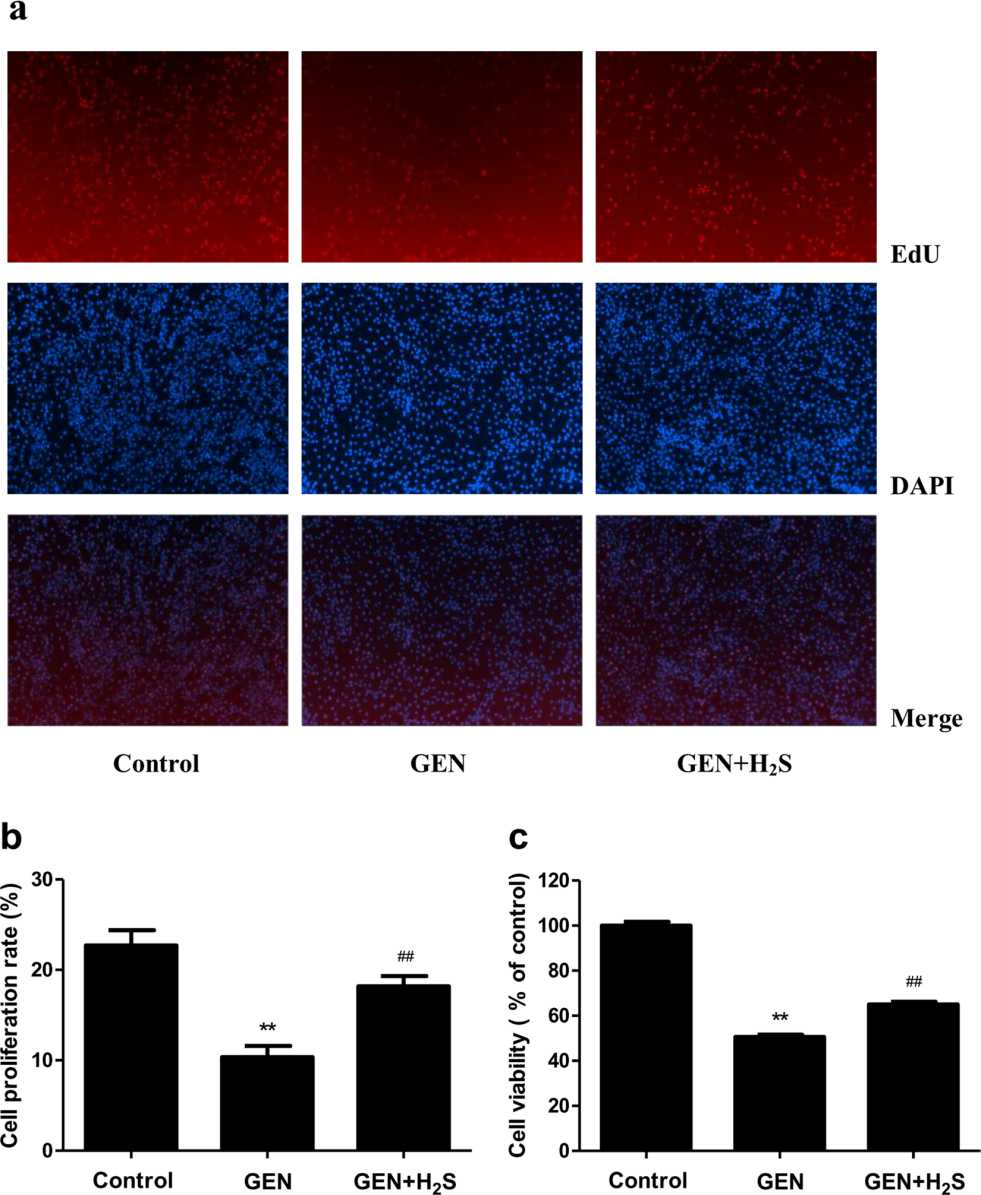
Effects of H_2_S on the viability and proliferation of the GEN-treated NRK-52E cells. (**a**) The cell proliferation was determined by EdU assay. The proliferative cell nuclei were stained by EdU assay with red, and all nuclei were stained by DAPI with blue (original magnification, ×100). (**b**) The cell proliferation rate was calculated. (**c**) The cell viability was detected by MTS assay. Values were presented as mean ± SEM (n = 6); *P < 0.05, **P < 0.01 compared with the control group; ^#^ *P* < 0.05, ^##^ *P* < 0.01 compared with the GEN group.

As revealed by the EdU assay, the incubation of NRK-52E cells with GEN (3 mM) led to a significant reduction in cell proliferation (Fig. 1a,b). By contrast, administration of H_2_S induced a significant increase in cell viability, compared with the GEN group. Furthermore, as shown in Fig. 1c, GEN decreased the cell viability, while H_2_S treatment significantly increased the cell viability. Collectively, these data demonstrate that H_2_S could effectively relieve GEN-induced cytotoxicity in NRK-52E cells.

### H_2_S protects NRK-52E cells from GEN-induced ROS production and apoptosis

**Figure 2:**
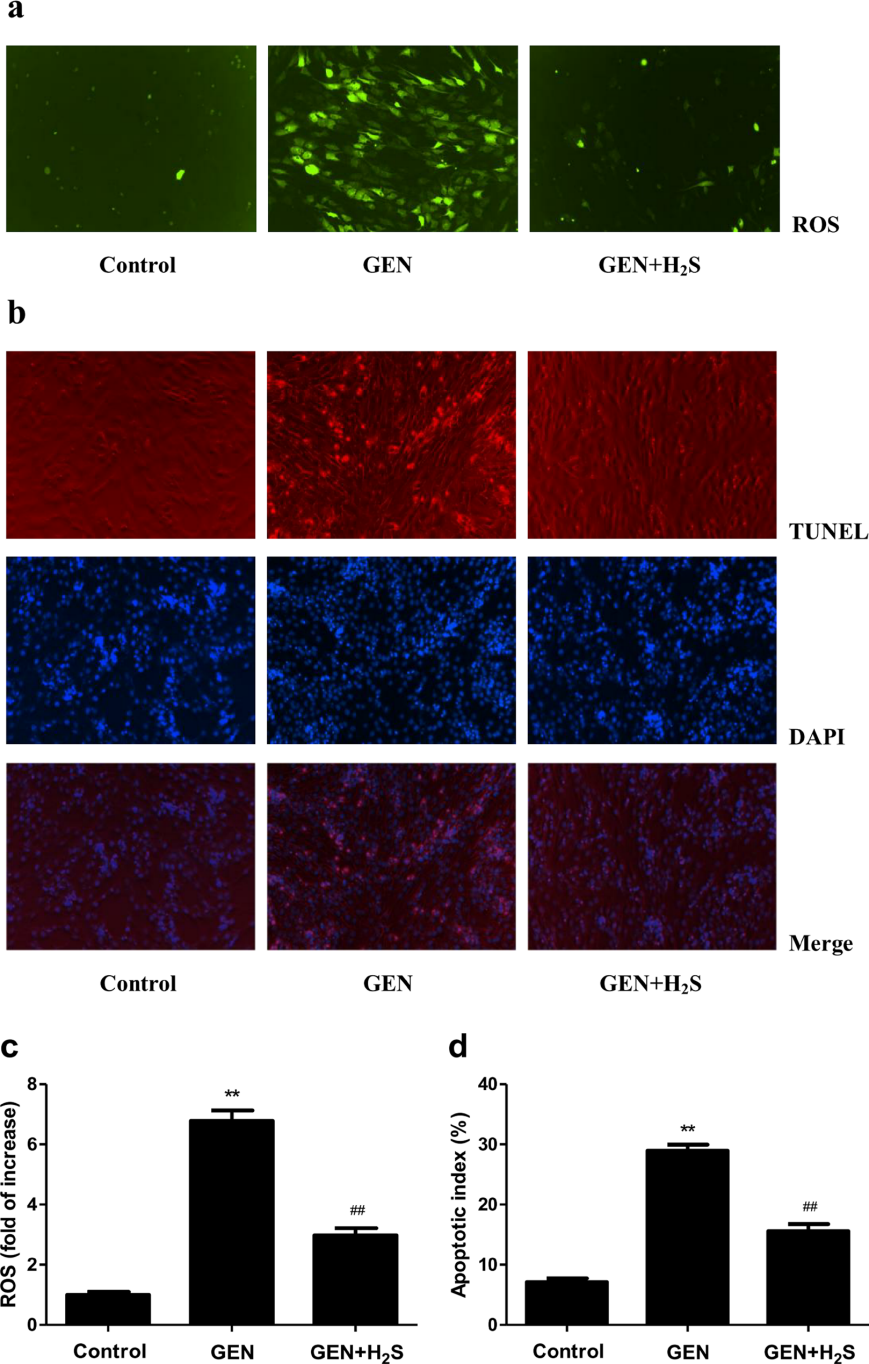
Effects of H_2_S on the intracellular ROS production and apoptosis in GEN-treated NRK-52E cells. (**a**) The intracellular ROS production was detected using the fluorescent probe DCF-DA (shown in green; original magnification, ×100). (**b**) The cell apoptosis was determined by TUNEL assay. Apoptotic cell nuclei were stained by TUNEL assay with red, and all nuclei were stained by DAPI with blue (original magnification, ×100). (**c**) The intracellular ROS production was measured. (**d**) The apoptotic index was calculated. Values were presented as mean ± SEM (n = 6); *P < 0.05, **P < 0.01 compared with the control group; ^#^ *P* < 0.05, ^##^ *P* < 0.01 compared with the GEN group.

Inducing apoptosis is a key nephrotoxic mechanism in GEN-treated NRK-52E cells^22^. ROS have been considered important mediators of GEN-induced apoptosis. ROS generation is often involved in the mitochondrion-mediated signaling pathway of apoptosis^29^. Compared with the control group, ROS generation increased 5.8- fold in GEN group, and H_2_S significantly reduced ROS generation by 3.8-fold compared with the GEN group (Fig. 2a,c). In addition, apoptotic index increased 21.8% in GEN group compared with the control group and decreased 13.4% in the GEN+H_2_S group compared with the GEN group (Fig. 2b,d). These results indicate that H_2_S could alleviate GEN-induced nephrotoxicity by reducing ROS production and apoptosis in NRK-52E cells.

### H_2_S improves general status of CRF rats

**Figure 3:**
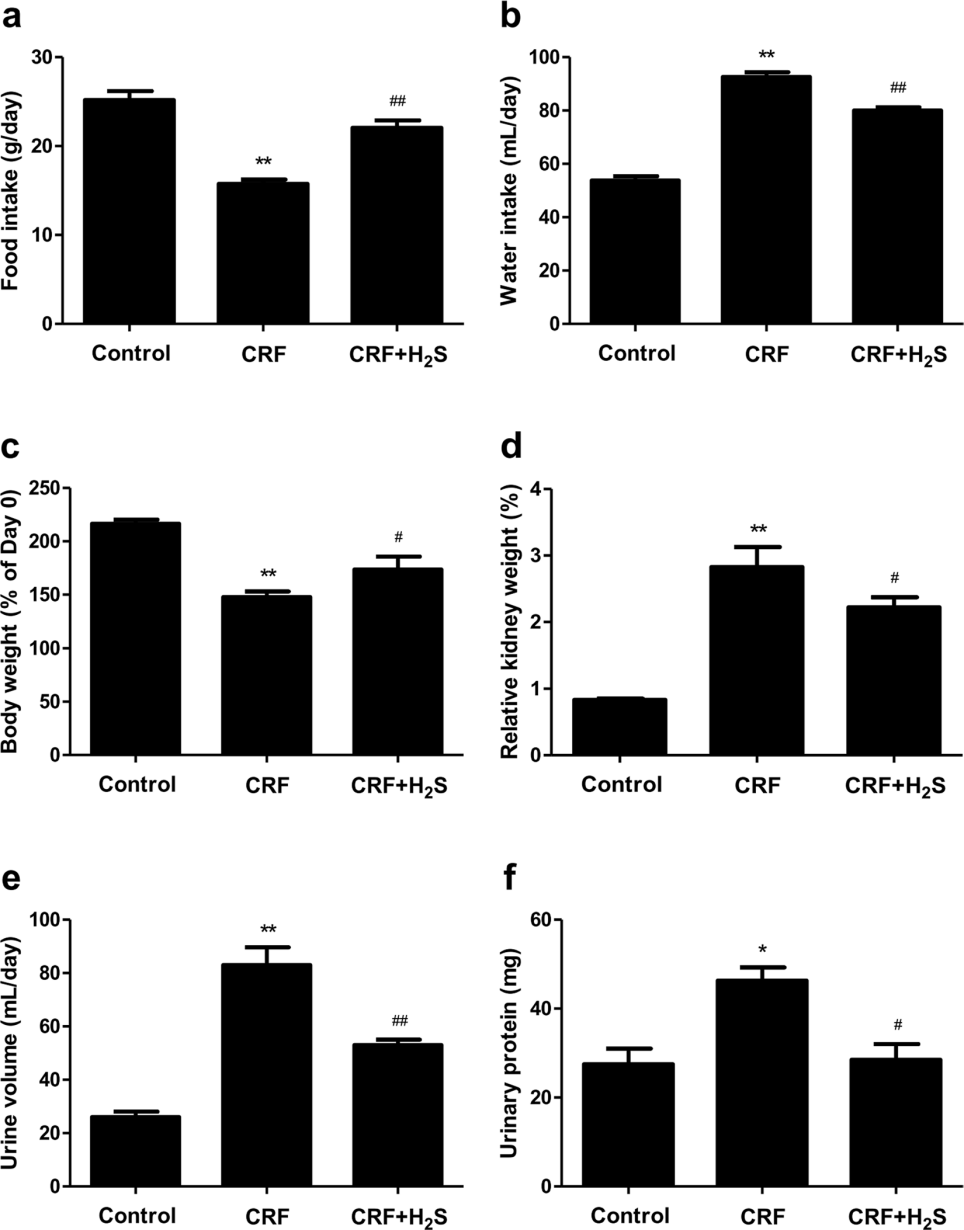
Effects of H_2_S on the physiological parameters in CRF rats. (**a**) Food intake. (**b**) Water intake. (**c**) The body weight change of rats. (**d**) Relative kidney weight of rats. (**e**) Urine volume. (**f**) Urinary protein. Values were presented as mean ± SEM (n = 8); *P < 0.05, **P < 0.01 compared with the control group; ^#^ *P* < 0.05, ^##^ *P* < 0.01 compared with the CRF group.

As shown in Fig. 3a,b, in comparison with the control group, CRF rats exhibited decreased food intake and increased water intake, H_2_S treatment significantly reversed these changes. A trend in the decrease of body weight in CRF rats, which was altered by H_2_S, has been observed (Fig. 3c). In addition, CRF rats showed increased relative kidney weight, urine volume, and urinary protein when compared with the control group, which were dramatically reversed by H_2_S treatment (Fig. 3d–f). In sum, these results show that H_2_S could significantly improve the general status of the rats with adenine-induced renal damage.

### H_2_S enhances the kidney function of CRF rats

**Figure 4:**
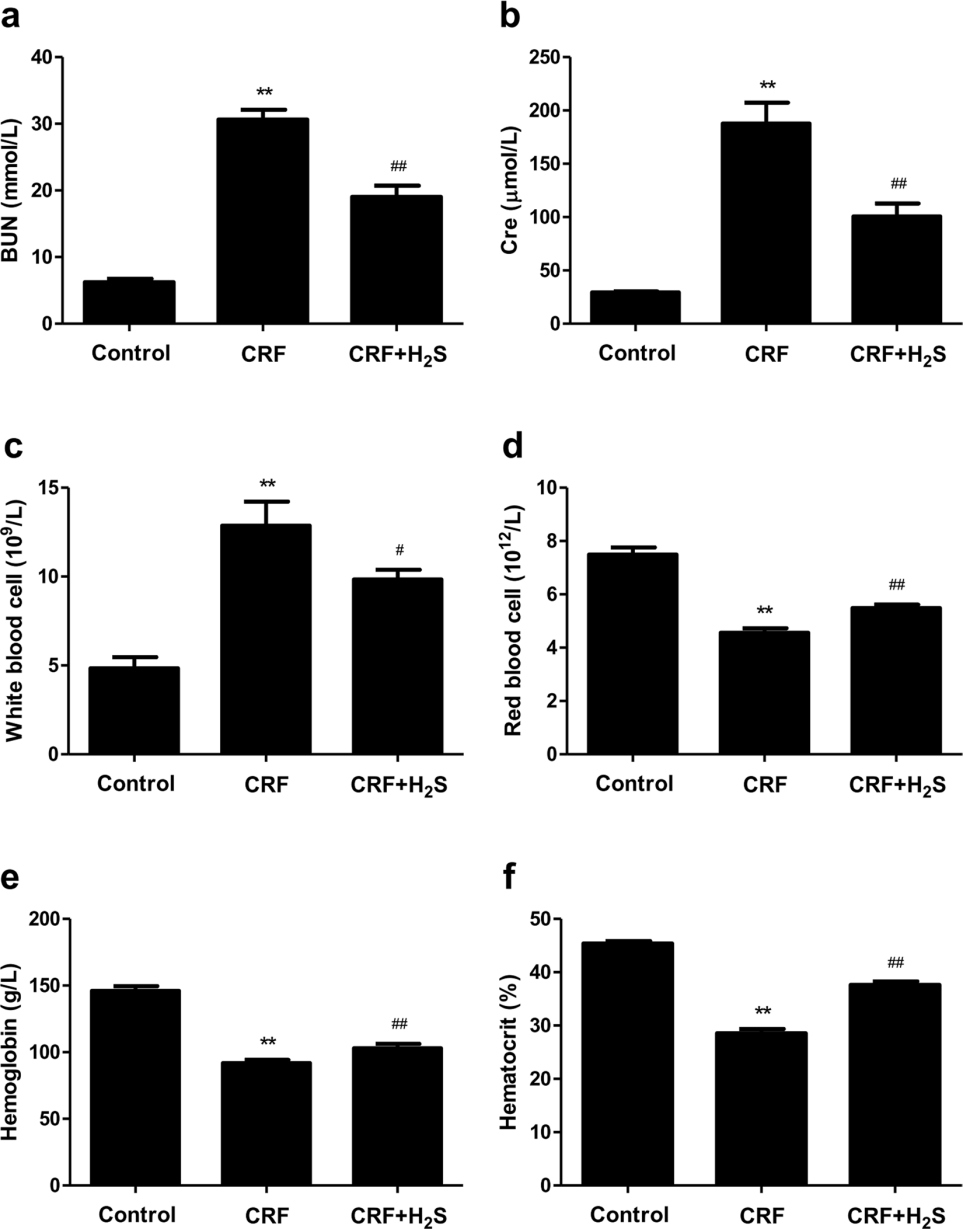
Effects of H_2_S on the kidney function and blood routine parameters in CRF rats. (**a**) BUN. (**b**) Cre. (**c**) White blood cell. (**d**) Red blood cell. (**e**) Hemoglobin. (**f**) Hematocrit. Values were presented as mean ± SEM (n = 8); **P < 0.01 compared with the control group; ^#^ *P* < 0.05, ^##^ *P* < 0.01 compared with the CRF group.

Plasma BUN and Cre are the most commonly used markers of kidney function in clinical practice^30, 31^. The concentrations of BUN and Cre and WBC count were significantly increased whereas HGB, HCT, and RBC count were dramatically decreased in CRF group compared to the control group (Fig. 4). These results indicate that the rat model exhibited typical pathologic features associated with CRF. Compared with the CRF group, the CRF+H_2_S group showed remarkably lower Cre and BUN levels and WBC count and significantly higher HGB, HCT, and RBC count (Fig. 4). These results demonstrate that the kidney function of CRF rats could be effectively enhanced by administration of H_2_S.

### H_2_S ameliorates renal injury in CRF rats

**Figure 5:**
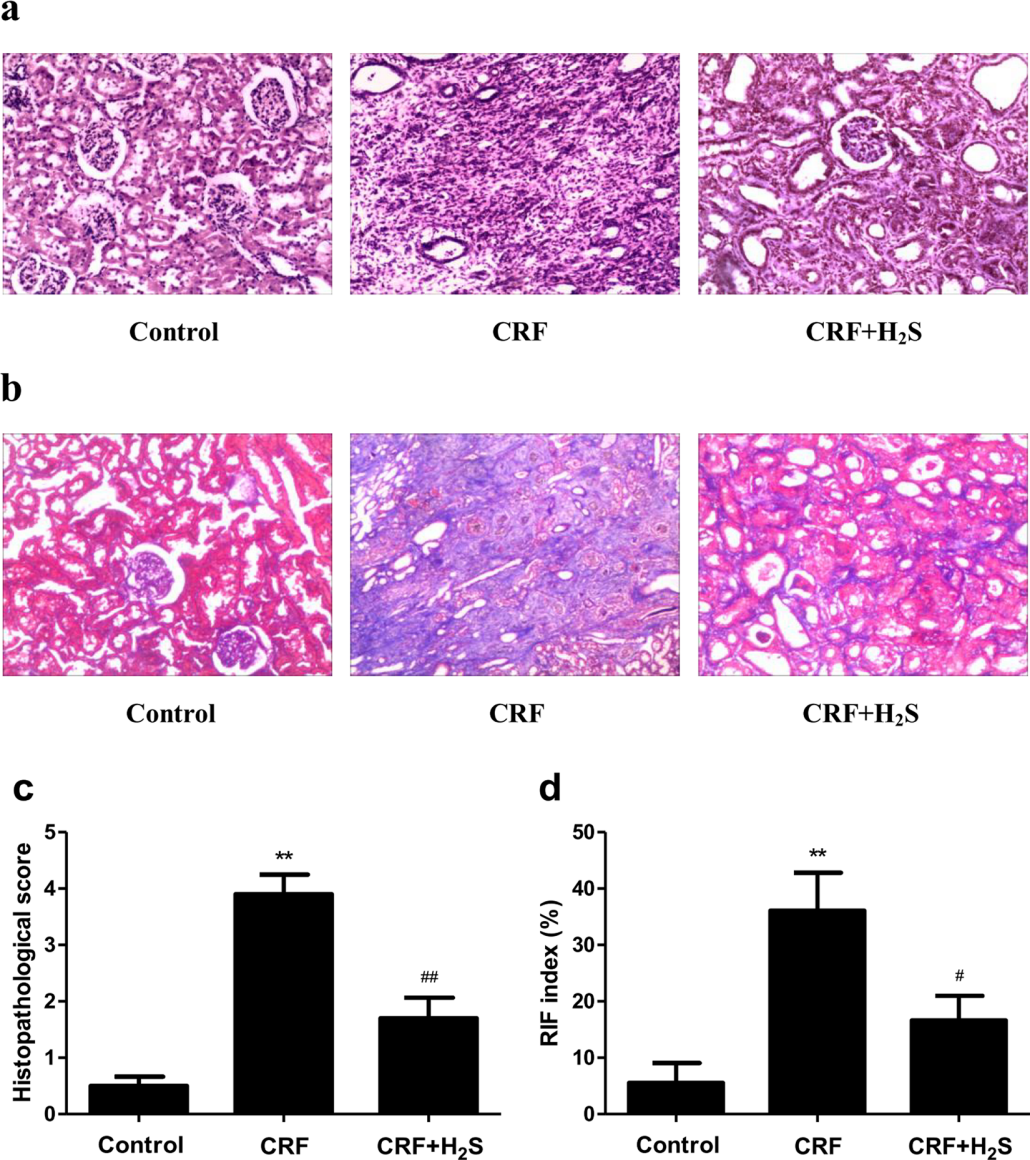
Effects of H_2_S on morphological changes in the kidney of CRF rats. (**a**) The kidney pathological changes were detected by HE staining (original magnification, ×100). (**b**) The tubulointerstitial fibrosis was detected by Masson staining (original magnification, ×100). (**c**) The histopathological score was calculated. (**d**) The extent of the renal lesions was represented by the RIF index. Values were presented as mean ± SEM (*n* = 6); **P < 0.01 compared with the control group; ^#^ *P* < 0.05, ^##^ *P* < 0.01 compared with the CRF group.

Figure 5 showed representative photomicrographs of the HE and MT stainings of the kidney tissues from the control, CRF, and CRF+H_2_S groups. There were no signs of damage in the control group. The kidney tissues of CRF rats showed severe renal injury marked by severe interstitial inflammatory cell infiltration, tubular dilation and atrophy, as well as fibrosis. These results indicate that the rat model exhibited the typical pathological features associated with CRF, which were consistent with previous studies^32, 33^. The severity of renal injury in rats with CRF was significantly ameliorated by treatment with H_2_S.

### H_2_S reduces apoptosis level in the kidney of CRF rats

**Figure 6:**
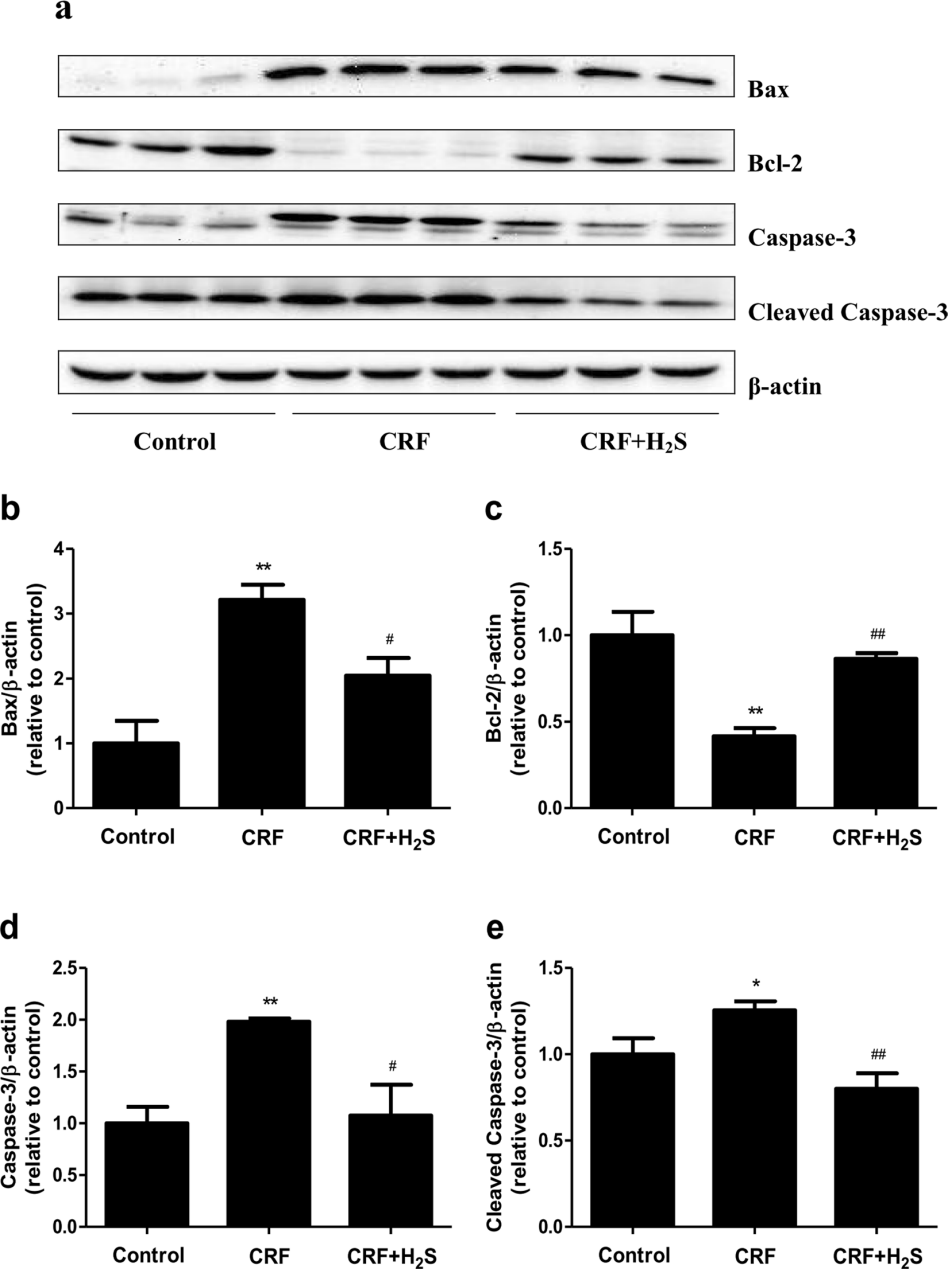
Effects of H_2_S on the protein expression of Bax, Bcl-2, Caspase-3, and Cleaved Caspase-3 in the kidney of CRF rats were measured. (a) The expression levels of Bax, Bcl-2, Caspase-3, and Cleaved Caspase-3 were detected by Western blot. β-actin was used as an internal control. Bar graphs showed the quantification of Bax (b), Bcl-2 (c), Caspase-3 (d), and Cleaved Caspase-3 (e). Values were presented as mean ± SEM (n = 3); *P < 0.05, **P < 0.01 compared with the control group; ^#^ *P* < 0.05, ^##^ *P* < 0.01 compared with the CRF group.

Cell apoptosis can be widely detected in CRF patients and inhibition of apoptosis could delay the progress of CRF and reduce the occurrence of related complications^7^. Compared to the control group, the protein expression levels of Bax, Caspase-3, and Cleaved-caspase-3 were dramatically increased in the kidney of CRF rats (Fig. 6a,b,d,e). In contrast, the protein expression of Bcl-2 in CRF group was significantly lower than that in the control group (Fig. 6a,c). The protein expressions of Bax, Caspase-3, and Cleaved-caspase-3 were remarkably reduced in CRF+H_2_S group, in comparison with CRF group (Fig. 6a,b,d,e). In addition, the protein expression of Bcl-2 increased significantly in CRF+H_2_S group (Fig. 6a,c). These findings suggest that the apoptosis level is increased in the kidney of CRF rats, which could be reversed by administration of H_2_S.

### H_2_S abates oxidative stress in the kidney of CRF rats

**Figure 7:**
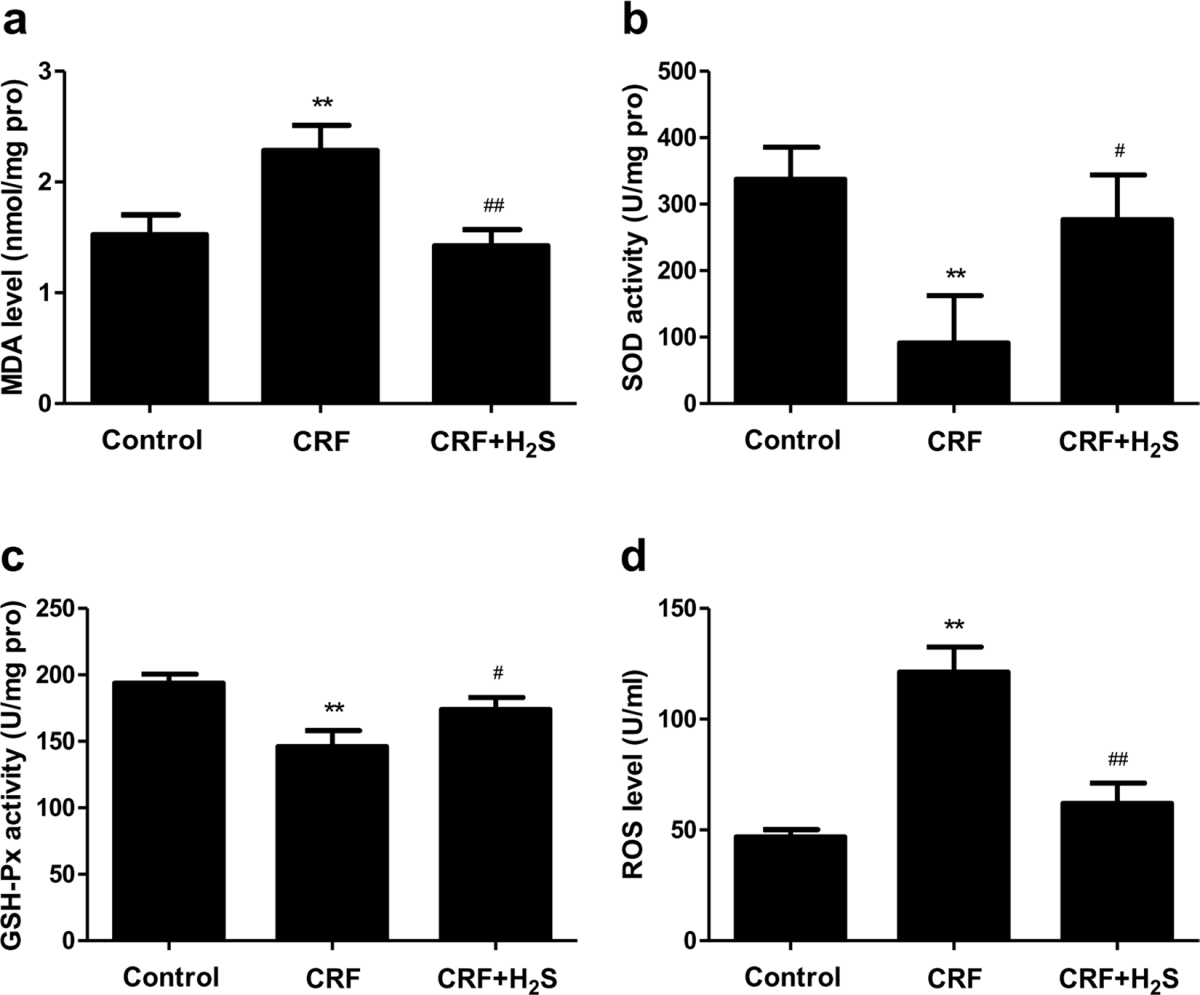
Effects of H_2_S on the MDA level, SOD and GSH-Px activities, and the ROS generation in the kidney of CRF rats. (**a**) MDA level. (**b**) SOD activity. (**c**) GSH-Px activity. (**d**) ROS level. Values were presented as mean ± SEM (n = 8); *P < 0.05, **P < 0.01 compared with the control group; ^#^ *P* < 0.05, ^##^ *P* < 0.01 compared with the CRF group.

A recent study showed that adenine treatment significantly depressed total antioxidant capacity in the kidney of rats^33^. To observe the effect of H_2_S on oxidative stress induced by adenine, the activities of antioxidant enzymes, MDA generation, and ROS accumulation were determined. As shown in Fig. 7, the levels of MDA and ROS were markedly increased, and the activities of SOD and GSH-Px were significantly decreased compared with the control group, which were all reversed by treatment with H_2_S. These results indicate that H_2_S could abate adenine-induced oxidative stress in the kidney of CRF rats.

### H_2_S abolishes the phosphorylation of mitogen-activated protein kinases (MAPKs) in the kidney of CRF rats

**Figure 8:**
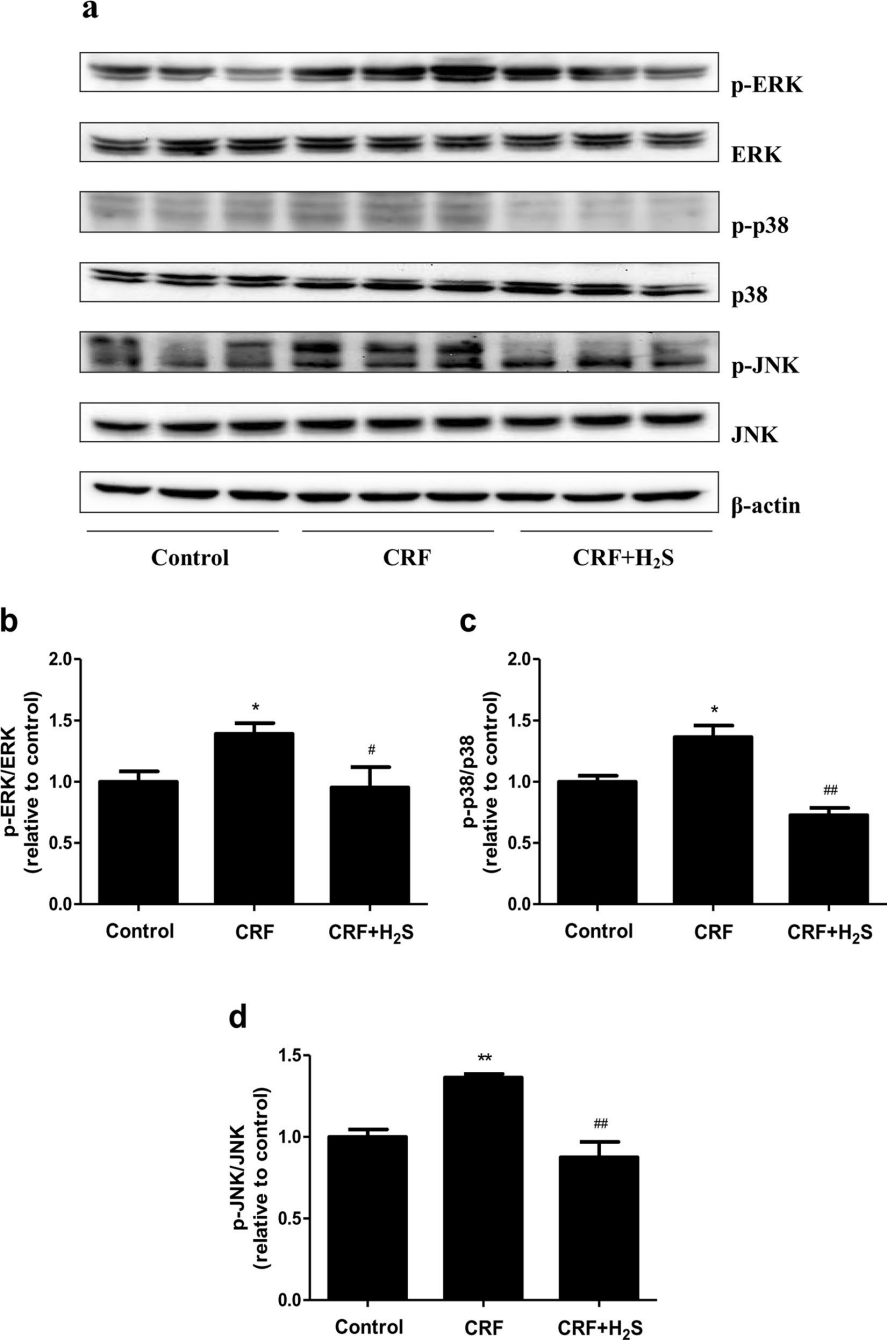
Effects of H_2_S on the MAPK pathway in the kidney of CRF rats. (**a**) The expression levels of p-ERK, ERK, p-p38, p38, p-JNK, and JNK were detected by Western blot. β-actin was used as an internal control. Bar graphs showed the quantification of p-ERK/ERK (**b**), p-p38/p38 (**c**), and p-JNK/JNK (**d**). Values were presented as mean ± SEM (n = 3); *P < 0.05, **P < 0.01 compared with the control group; ^#^ *P* < 0.05, ^##^ *P* < 0.01 compared with the CRF group.

MAPK signaling pathway mediates a number of cellular activities in response to extracellular stimuli such as heat and stress^34^. ERK1/2, JNK, and p38 are three major components of MAPK which play important roles in cell migration and apoptosis^35, 36^. As shown in Fig. 8, CRF triggered the phosphorylation of p38, JNK, ERK with distinct patterns. However, administration of H_2_S significantly abolished the increase of MAPKs phosphorylation induced by CRF, suggesting that H_2_S could reduce the apoptosis level in the kidney of CRF rats through MAPK signaling pathway.

### H_2_S alleviates renal inflammation in CRF rats

**Figure 9:**
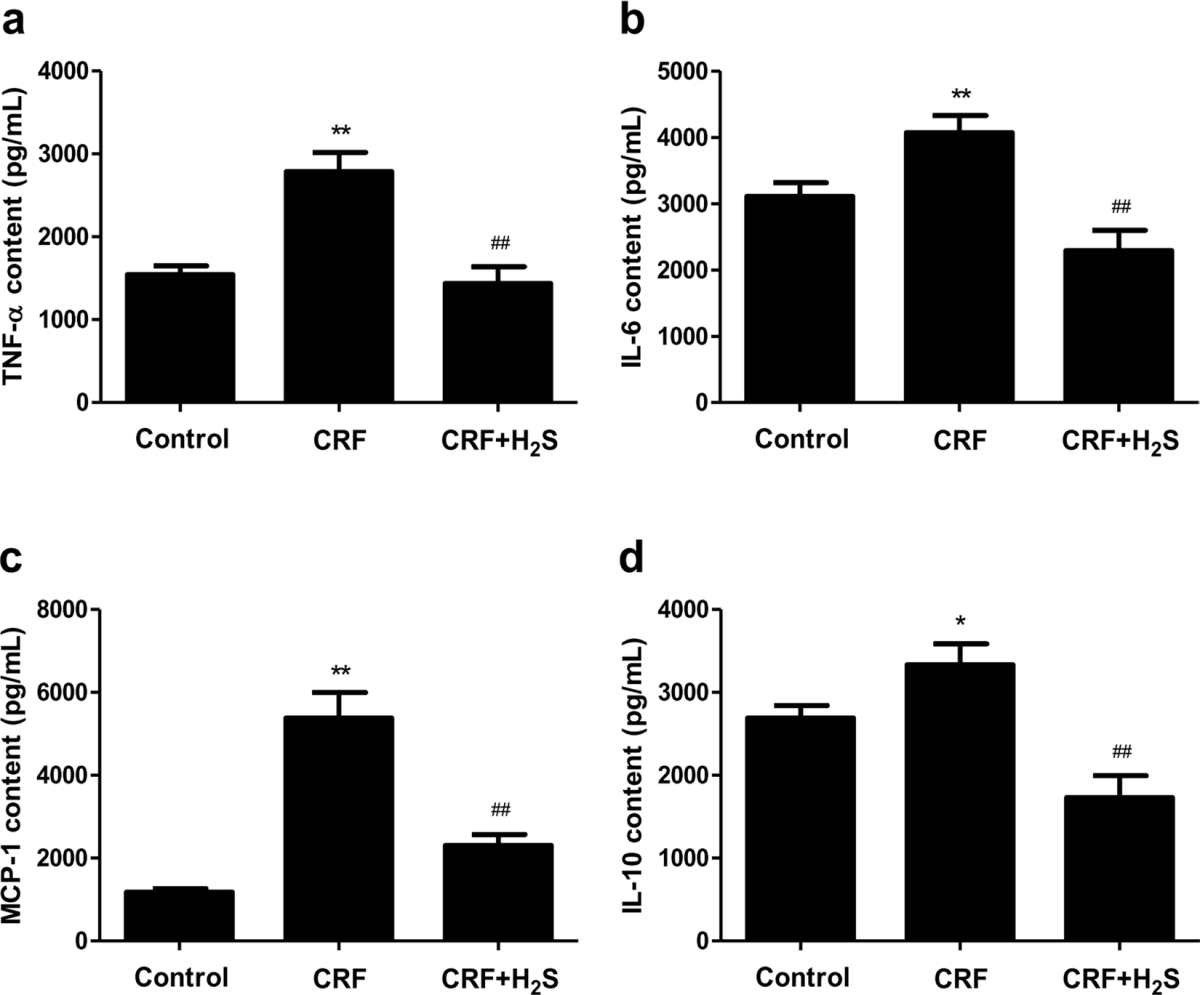
Effects of H_2_S on the cytokine levels in the kidney of CRF rats were assayed using ELISA techniques. The expression levels of TNF-α (**a**), IL-6 (**b**), MCP-1 (**c**), and IL-10 (**d**) were measured. Values were presented as mean ± SEM (n = 8); *P < 0.05, **P < 0.01 compared with the control group; ^##^ *P* < 0.01 compared with the CRF group.

Adenine treatment could induce a highly significant increase in plasma concentrations of some inflammatory cytokines, such as TNF–α and interleukin-1 beta (IL-1β)^33^. Whether H_2_S could reduce renal inflammation in CRF rats remains unknown. In this study, the inflammatory cytokine levels in the kidney were determined using ELISA techniques. Compared with the control group, the expression levels of TNF-α, IL-6, IL-10, and MCP-1 were significantly increased. Treatment with H_2_S remarkably decreased the levels of TNF-α, IL-6, IL-10, and MCP-1 (Fig. 9), suggesting that H_2_S could effectively alleviate renal inflammation in CRF rats.

### H_2_S decreases the expression of NF-*κ*B in the kidney of CRF rats

**Figure 10:**
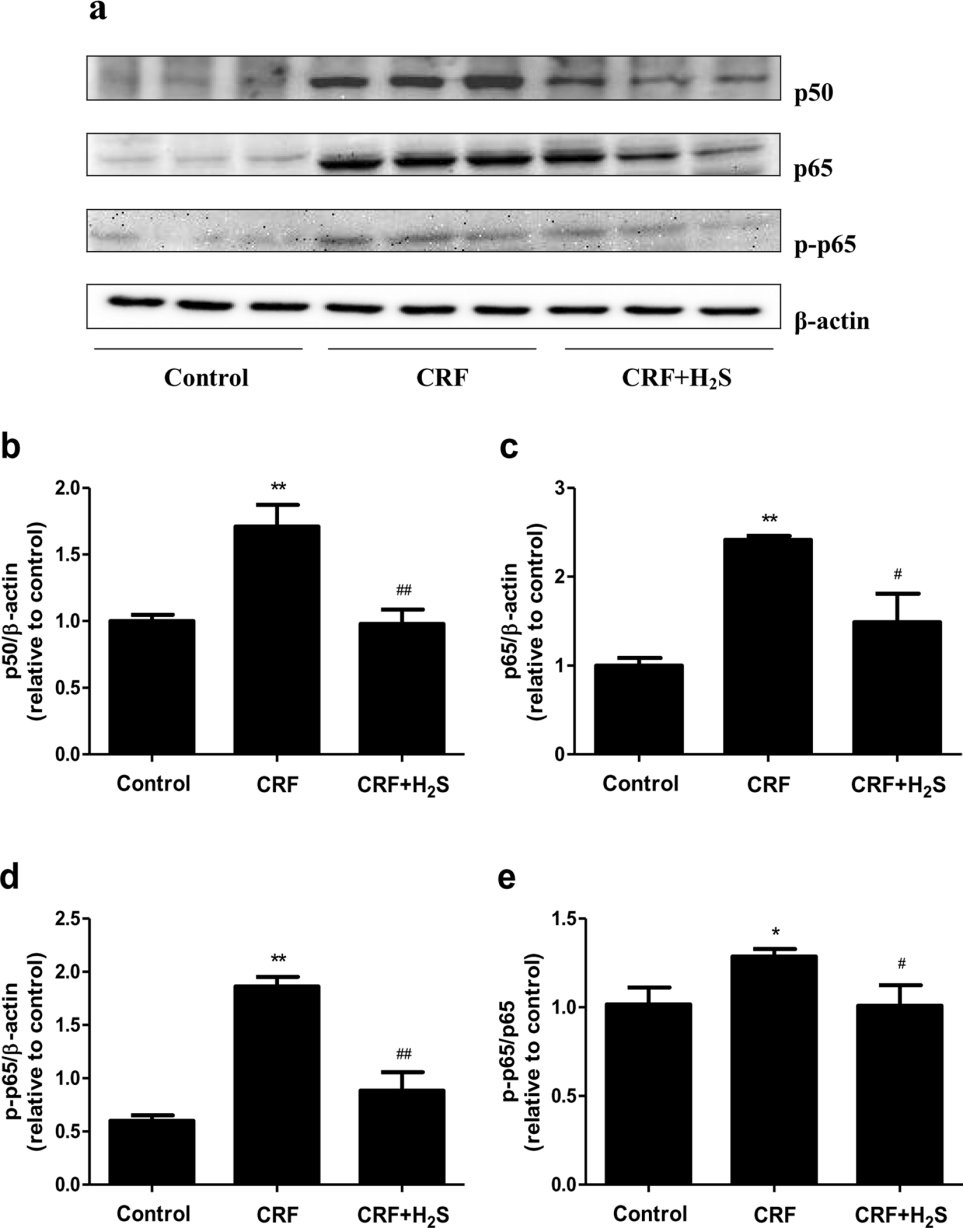
Effects of H_2_S on the NF-κB pathway in the kidney of CRF rats. (**a**) The expression levels of p50, p65, and p-p65 were detected by Western blot. β-actin was used as an internal control. Bar graphs showed the quantification of p50 (**b**), p65 (**c**), p-p65 (**d**), and p-p65/p65 (**e**). Values were presented as mean ± SEM (n = 3); *P < 0.05, **P < 0.01 compared with the control group; ^#^ *P* < 0.05, ^##^ *P* < 0.01 compared with the CRF group.

NF-*κ*B is a transcription factor that plays an important role in regulating the expression of cytokine genes involved in several inflammatory diseases, including diabetes, atherosclerosis, and metabolic syndrome^37^. The most abundant form of NF-κB is the heterodimer composed of p50 and p65^38, 39^. In the present study, the protein expressions of p50, p65, and p-p65 in the kidney of rats were measured to investigate the underlying mechanism of H_2_S on the cytokine regulation. Compared with the control group, the protein expressions of p50, p65, and p-p65 and the p-p65/p65 ratio were significantly increased (Fig. 10). Treatment with H_2_S remarkably decreased the expression levels of p50, p65, and p-p65, as well as the ratio of p-p65/p65 in the kidney of CRF rats (Fig. 10), indicating that H_2_S could reduce kidney inflammation induced by CRF through the down-regulation of NF-*κ*B expression.

## Discussion

H_2_S has recently been recognized as an endogenous gaseous signaling molecule, along with nitric oxide and carbon monoxide^8–10, 15, 28^. A growing body of evidence indicates that H_2_S plays important and complex roles in renal physiological and pathophysiological processes^11, 13, 14^. The NRK-52E cell line has been widely used in *in vitro* models for studying GEN-induced nephrotoxicity^20–22^. Our results showed that H_2_S treatment significantly increased the viability and proliferation of the GEN-treated NRK-52E cells. Inducing apoptosis is an important nephrotoxic mechanism of GEN in NRK-52E cells^22^. The results indicated that H_2_S dramatically decreased the apoptotic index in GEN-treated NRK-52E cells. An increasing number of evidence indicates that ROS are important mediators of GEN-induced apoptosis^20^. H_2_S treatment significantly reduced ROS generation in GEN-treated NRK-52E cells. These results together suggest that H_2_S could alleviate GEN-induced nephrotoxicity by reducing ROS-mediated apoptosis in NRK-52E cells.

Currently, there are two experimental animal models for CRF, namely the chemical model (using adenine in the food) and the surgical model (5/6 remnant kidney model, or renal mass reduction model)^23^. Adenine-induced CRF avoids the potential complications of alternative techniques that require surgery to induce chronic kidney disease^40^. In addition, this method produces more pronounced reductions in glomerular filtration rate compared with the model of 5/6 nephrectomy^41^. Thus, adenine-induced CRF in rats was adopted as a disease model for the evaluation of the effect of H_2_S on CRF. The results showed that the rats fed on the adenine diet for 4 weeks showed increased water intake, urine production, urinary protein, and relative kidney weight, which were similar to the signs and symptoms in rats with adenine-induced CRF^41, 42^. In addition, a previous study has reported a reduction in body weight in adenine-fed rats which could be attributed to the reduced food consumption^43^. Our results were in good accordance with the findings. Administration of H_2_S effectively ameliorated all the above adenine-induced changes.

BUN and Cre are the most commonly used markers for detecting nephrotoxicity in traditional clinical pathology^44^. Treatment with H_2_S significantly decreased the levels of BUN and Cre, suggesting that H_2_S could reduce nephrotoxicity in CRF rats. Another common complication of CRF is anemia which often contributes to poor functional status and quality of life for CRF patients^40^. The RBC, HGB and HCT values of CRF rats were significantly lower than those of the control group, confirming that anemia had developed in our animal model of CRF. Our results showed that H_2_S was able to reduce the extent of anemia observed in rats with CRF, which was in line with a recent study^45^.

Apoptosis is an intrinsic cell-suicide program that is critical for the normal development and maintenance of tissue homeostasis in multicellular organisms^46^. There are two main apoptotic signaling pathways: the death receptor-mediated extrinsic pathway and the mitochondria-mediated intrinsic pathway^47^. The proteins of the Bcl-2 family are key regulators of the mitochondrial pathway, including pro-apoptotic members (such as Bax) and anti-apoptotic members (such as Bcl-2), which can regulate the activation of caspases that cleave a number of cellular proteins, such as caspase-3^47, 48^. Recent studies found that CRF rats had increased apoptosis levels, up-regulated Bax expression, and down-regulated Bcl-2 expression in renal tissues^48, 49^. In line with the above findings, our results showed that the expression levels of Bax, Caspase-3, and Cleaved Caspase-3 were significantly increased and the expression level of Bcl-2 was dramatically decreased in CRF rats. Treatment with H_2_S remarkably decreased the levels of Bax, Caspase-3, and Cleaved Caspase-3, whereas it increased kidney Bcl-2 expression in CRF rats, suggesting that H_2_S could effectively reduce the apoptotic levels induced by CRF in rats.

It is widely accepted that relatively high level of ROS causes redox imbalance, induces cell apoptosis or necrosis during a wide variety of physiological and pathological conditions^47, 50, 51^. Our results indicated that the levels of ROS and MDA were markedly increased, and the activities of anti-oxidative enzymes, SOD and GSH-Px, were significantly decreased compared with the control group, suggesting that ROS could induce apoptosis in the kidney of CRF rats. All these changes were reversed by administration of H_2_S. Recent studies have proven that ROS can activate MAPKs and apoptotic cell death induced by ROS is mediated by MAPK pathway^52–54^. The present study identified that CRF increased the phosphorylation of p38, JNK, and ERK, whereas H_2_S treatment significantly reversed the CRF-induced increase in MAPKs phosphorylation. These results demonstrated that H_2_S was able to reduce the apoptotic levels induced by CRF through ROS-mediated MAPK pathway.

Inflammatory cytokines play important roles in the development and progression of CRF^55, 56^. It is well documented that the levels of several inflammatory cytokines were higher in CRF patients compared with control subjects, such as TNF-α, IL-6, IL-10, and MCP-1^55–57^. Similarly, our data indicated that the levels of these cytokines were significantly increased compared with the control group. High levels of TNF-α, IL-6, and MCP-1 indicated activation and increased production of cytokines, which can lead to an inflammatory state in the kidney of CRF rats. High levels of IL-10 in the kidney could be suggestive of an aberration in the pro anti-inflammatory adjustment. Administration of H_2_S effectively alleviated renal inflammation in CRF rats. The NF-*κ*B network is involved in a wide range of inflammatory, autoimmune, and malignant disorders^37, 58, 59^. The p50/p65 heterodimer is considered the most important transcription factor of the NF-*κ*B pathway and is specifically referred to as NF-*κ*B^59, 60^. A recent study indicated that the expression of NF-*κ*B was upregulated in the kidney of CRF rats^61^. Our results showed that CRF increased kidney p50, p65, and p-p65 protein expressions as well as the p-p65/p65 ratio, suggesting that CRF induced an inflammatory state in the kidney of rats. However, treatment with H_2_S significantly reversed the changes induced by CRF, suggesting that H_2_S could reduce kidney inflammation through the downregulation of NF-*κ*B expression.

**Figure 11:**
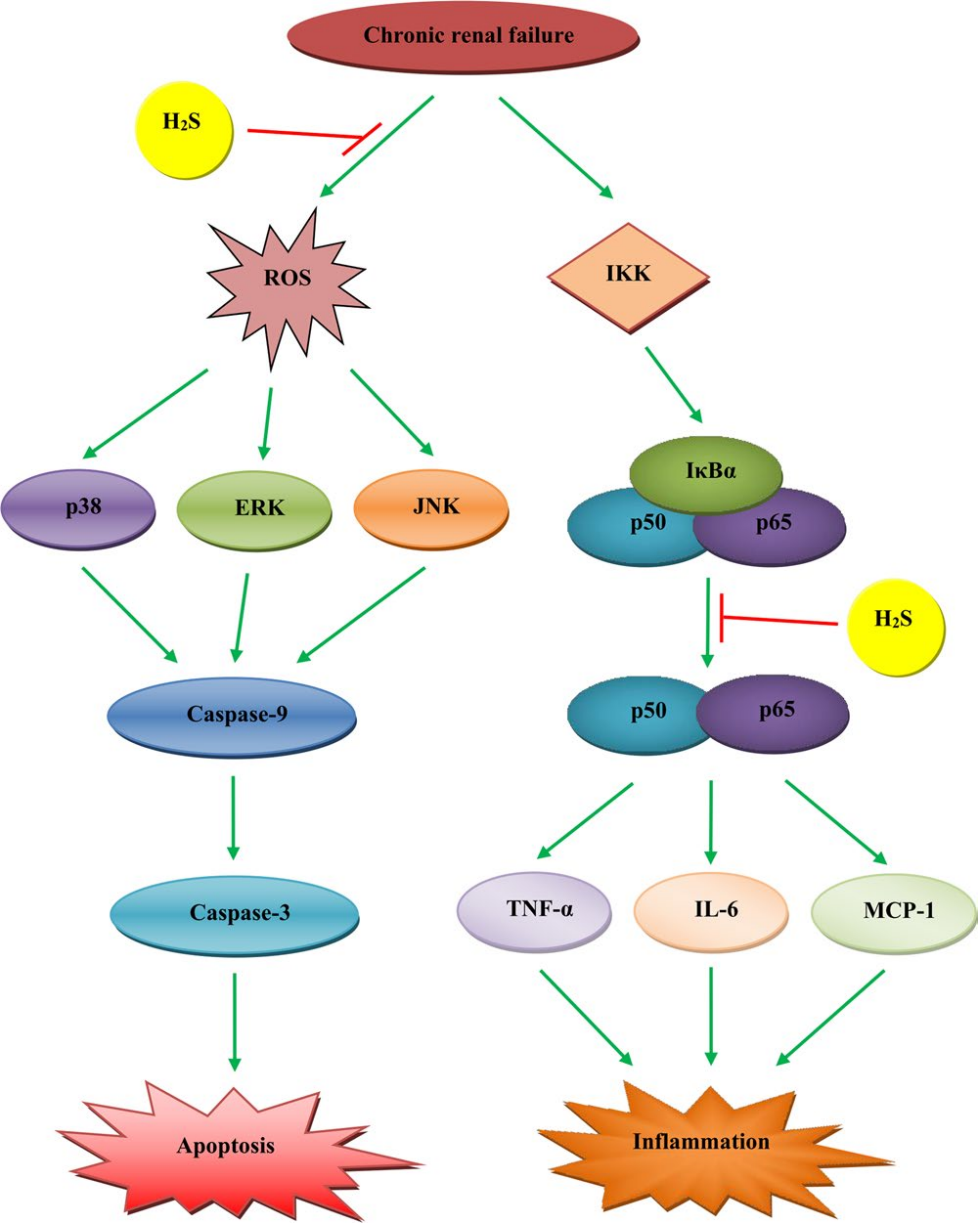
A schematic illustration of the role of H_2_S in ameliorating CRF. H_2_S could ameliorate CRF in rats by inhibiting apoptosis and inflammation through the ROS/MAPK and NF-*κ*B signaling pathways. IKK, inhibitor kappa B kinase; IκBα, inhibitor kappa B-alpha.

In conclusion, our results demonstrate that H_2_S is able to ameliorate CRF in rats by inhibiting apoptosis and inflammation through the ROS/MAPK and NF-*κ*B signaling pathways (Fig. 11). Therefore, H_2_S or its releasing compounds may serve as a potential therapeutic molecule for CRF.

## Electronic supplementary material

Supplementary material
