# Supplementary material for: Hydrogen sulfide ameliorates chronic renal failure in rats by inhibiting apoptosis and inflammation through ROS/MAPK and NF-κB signaling pathways
Source: Sci Rep. 2017 Mar 28;7:455. doi: 10.1038/s41598-017-00557-2 (PMC5428696; doi:10.1038/s41598-017-00557-2)
Supplement: Supplementary file 1 — Supplementary material [file 41598_2017_557_MOESM1_ESM.pdf]

# Hydrogen sulfide ameliorates chronic renal failure in rats by inhibiting apoptosis and inflammation through ROS/MAPK and NF-$\kappa$B signaling pathways

**Dongdong Wu [a,1], Ning Luo [b,1], Lianqu Wang [b], Zhijun Zhao [c], Hongmin Bu [b], Guoliang Xu [b], Yongjun Yan [b], Xinping Che [b], Zhiling Jiao [b], Tengfu Zhao [b], Jingtao Chen [b], Ailing Ji [a], Yanzhang Li [a,]*, Garrick D. Lee [b,]***

[a] *Henan University School of Medicine, Kaifeng 475004, Henan, China*

[b] *The First Affiliated Hospital of Henan University, Kaifeng 475001, Henan, China*

[c] *Luohe Medical College, Luohe 462002, Henan, China*

[1]Dongdong Wu and Ning Luo contributed equally to this work and should be regarded as co-first authors.

***** Corresponding authors: The First Affiliated Hospital of Henan University, Kaifeng 475001, Henan, China. Tel: +86 371 22735069; Fax: +86 371 25661846 (G. Lee). Henan University School of Medicine, Kaifeng 475004, Henan, China. Tel: +86 371 23880585; Fax: +86 371 23880585 (Y. Li).

*E-mail addresses:* Garricklee@foxmail.com (G. Lee), yanzhang206@163.com (Y. Li).

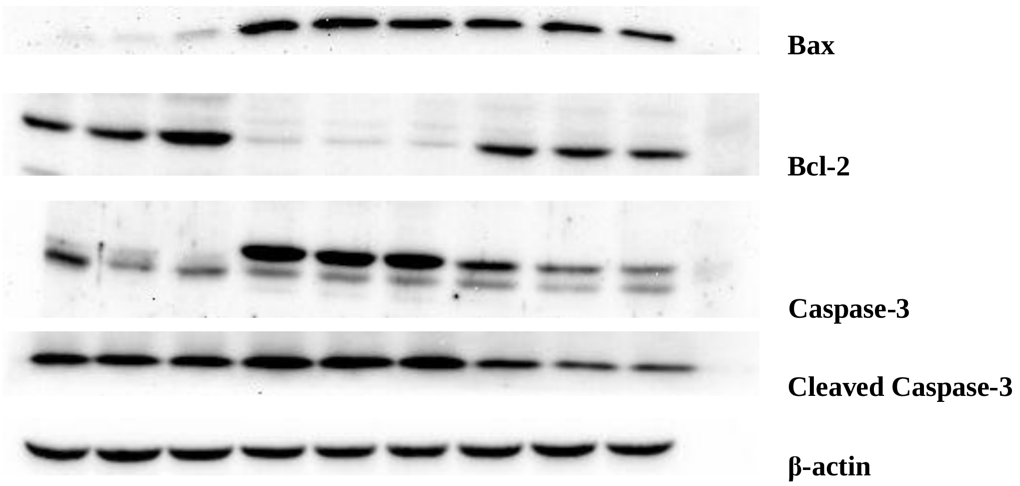
Bax
Bcl-2
Caspase-3
Cleaved Caspase-3
β-actin

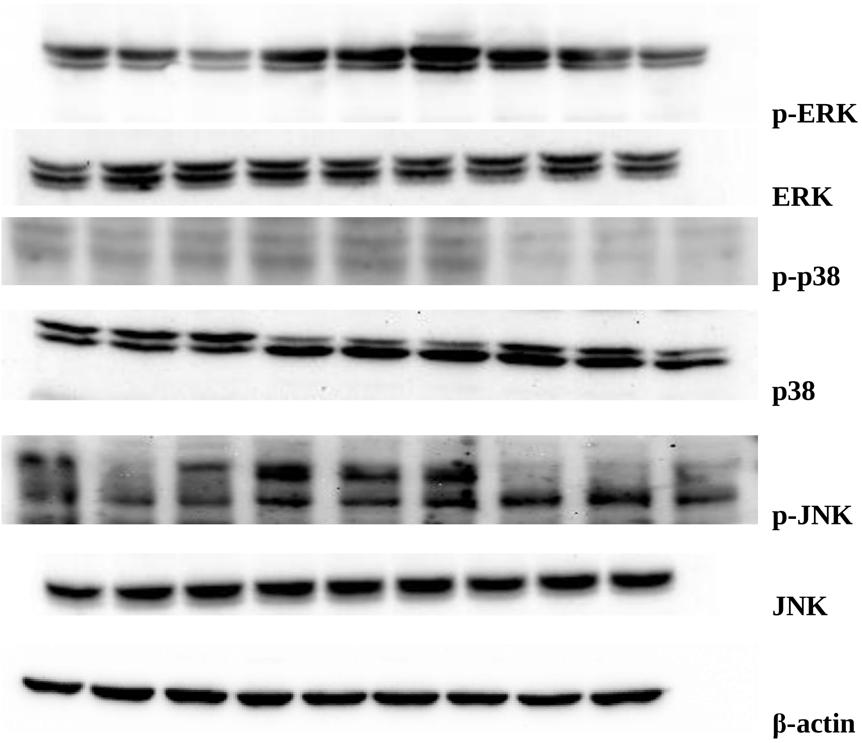
p-ERK
ERK
p-p38
p38
p-JNK
JNK
β-actin

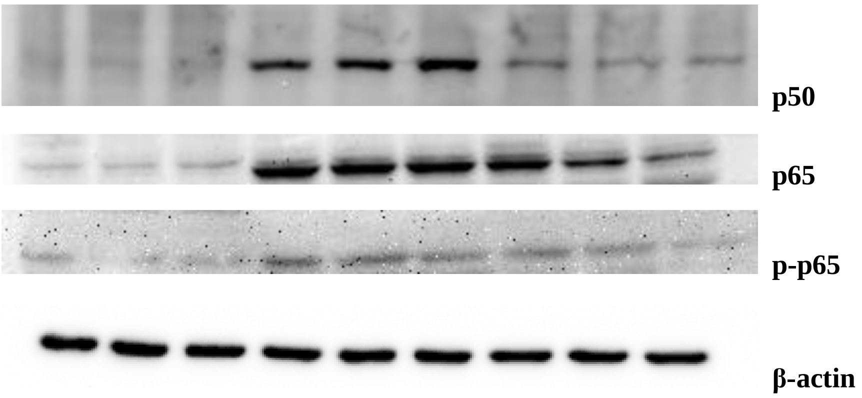
p50
p65
p-p65
β-actin
